# A Japanese prospective multi-institutional feasibility study on accelerated partial breast irradiation using interstitial brachytherapy: treatment planning and quality assurance

**DOI:** 10.1186/s13014-015-0430-8

**Published:** 2015-06-04

**Authors:** Yuki Otani, Takayuki Nose, Takushi Dokiya, Toshiaki Saeki, Yu Kumazaki, Shuuji Asahi, Iwao Tsukiyama, Ichirou Fukuda, Hiroshi Sekine, Naoto Shikama, Takao Takahashi, Ken Yoshida, Tadayuki Kotsuma, Norikazu Masuda, Eisaku Yoden, Kazutaka Nakashima, Taisei Matsumura, Shino Nakagawa, Seiji Tachiiri, Yoshio Moriguchi, Jun Itami, Masahiko Oguchi

**Affiliations:** Department of Radiation Oncology, Osaka University Graduate School of Medicine, 2-2 Yamadaoka, Suitashi, Osaka 565-0871 Japan; Department of Radiation Oncology, Nippon Medical School, Tamanagayama Hospital, Tama, Tokyo Japan; Department of Radiation Oncology, Kyoundo Hospital, Sasaki-Foundation, Chiyodaku, Tokyo Japan; Department of Breast Oncology, Saitama Medical University, International Medical Center, Hidaka, Saitama Japan; Department of Radiation Oncology, Saitama Medical University, International Medical Center, Hidaka, Saitama Japan; Department of Surgery, Aidu Chuo Hospital, Aiduwakamatsu, Fukushima Japan; Head of research institute for radiotherapy Southern TOHOKU Research Institute for Neuroscience Southern TOHOKU General Hospital, Koriyama, Fukushima Japan; Department of Radiation Oncology, National Hospital Organization Disaster Medical Center, Tachikawa, Tokyo Japan; Department of Radiology and Radiotherapy, The Jikei University, Daisan Hospital, Komae, Tokyo Japan; Department of Radiation Oncology, Osaka Medical College, Takatsuki, Osaka Japan; Department of Radiation Oncology, National Hospital Organization Osaka National Hospital, Osaka, Japan; Department of Surgery, Breast Oncology, National Hospital Organization Osaka National Hospital, Osaka, Japan; Department of Radiation Oncology, Kawasaki Medical School, Kurashiki, Okayama Japan; Department of General Surgery, Kawasaki Medical School, Kurashiki, Okayama Japan; Department of Radiology, National Hospital Organization Kyushu Medical Center, Fukuoka, Fukuoka Japan; Department of Breast Surgery, National Hospital Organization Kyushu Medical Center, Fukuoka, Fukuoka Japan; Department of Radiation Oncology, Kyoto City Hospital, Kyoto, Kyoto Japan; Department of Breast Oncology, Kyoto City Hospital, Kyoto, Kyoto Japan; Department of Radiation Oncology, National Cancer Center Hospital, Tokyo, Japan; Department of Radiation Oncology, Cancer Institute Hospital, the Japanese Foundation for Cancer Research, Tokyo, Japan

**Keywords:** APBI, HDR, Brachytherapy

## Abstract

**Background:**

In Japan, breast-conserving surgery with closed cavity has generally been performed for breast cancer patients, and accelerated partial breast irradiation (APBI) is considered difficult because Asian females generally have smaller breast sizes than Western females. Therefore, common identification of target and treatment plan method in APBI is required. A prospective multicenter study was conducted in Japan to determine institutional compliance with APBI using high-dose-rate interstitial brachytherapy (ISBT) designed for Japanese female patients.

**Methods:**

For this study, 46 patients were recruited at eight institutions from January 2009 to December 2011. The reproducibility of the ISBT–APBI plan was evaluated using three criteria: (1) minimum clinical target volume dose with a clip dose ≥ 6 Gy/fraction, (2) irradiated volume constraint of 40-150 cm^3^, and (3) uniformity of dose distribution, expressed as the dose non-uniformity ratio (DNR, V150/V100) < 0.35. The ISBT–APBI plan for each patient was considered reproducible when all three criteria were met. When the number of non-reproducible patients was ≤ 4 at study completion, APBI at this institution was considered statistically reproducible.

**Results:**

Half of the patients (52 %) had a small bra size (A/B cup). The mean values of the dose-constrained parameters were as follows: Vref, 117 cm^3^ (range, 40-282), DNR, 0.30 (range, 0.22-0.51), and clip dose, 784 cGy (range, 469-3146). A total of 43/46 treatment plans were judged to be compliant and ISBT–APBI was concluded to be reproducible.

**Conclusions:**

This study showed that multi-institutional ISBT–APBI treatment plan was reproducible for small breast patient with closed cavity.

## Background

In Japan, the incidence and mortality rate of breast cancer is growing rapidly, and the annual number of cases is expected to rise above 50,000 by 2020 [1]. Furthermore, the peak age for breast cancer in Japan is 10 years younger than that in Europe and USA, and the disease rate in 45–50-year-old females is high [2].

Breast-conserving surgery followed by postoperative radiotherapy is the standard of care for early-stage breast carcinoma [3, 4]. Generally, postoperative irradiation by whole-breast irradiation (WBI) reduces the rate of ipsilateral breast recurrence by one-third [4, 5]. However, studies have also reported that WBI prevents recurrence only near the tumor bed [3, 4, 6]. Moreover, WBI raises the mortality rate by increasing the risk of cardiovascular adverse events [5]. Thus, localized irradiation of the tumor bed is preferable for patients at a low risk of recurrence.

Accelerated partial breast irradiation brachytherapy (APBI) delivers radiation near the tumor bed over a short period of time [7–11]. In Europe and the USA, successful phase I/II clinical trials were completed in the 1990s, and large-scale phase III clinical trials are currently underway [8, 10]. In contrast, APBI is rarely used in Japan. One reason for this is the difficulty in target identification, caused by oncoplastic surgery, which is generally adopted in Japan. Moreover, APBI is considered difficult because Asian females generally have smaller breast sizes than Western females.

To promote APBI using interstitial brachytherapy, developing target identification consensus and reproducibility of this treatment among institutions should be first confirmed prospectively. This study presents the creative method of clinical target volume (CTV) and dose limitation for Asian females. Then, the reproducibility of interstitial brachytherapy (ISBT)-APBI is evaluated in breast cancer patients on multi-institutional feasibility clinical trial in Japan.

## Methods

### Patient cohort

A multi-institutional clinical trial was conducted from October 2009 to December 2011 at eight institutions in Japan. In total, 46 female patients with breast cancer were recruited prospectively for this study to receive adjuvant radiotherapy by ISBT–APBI alone after breast-conserving surgery.

The Institutional Review Board (IRB) of each institution approved this study. Written informed consent for data acquisition was obtained from each patient.

### Surgery

All patients underwent breast-conserving surgery, defined as resection of the primary tumor with > 0 cm of microscopic free margin. During surgery, the walls of the excision cavity were marked with at least four clips on the superior, inferior, medial, and lateral margins of the tumor bed. The brachytherapy applicators were inserted within 2 months after the breast-conserving surgery. All needles were inserted under image guidance by computed tomography (CT) or ultrasonography (US). More two-plane implants were made. The needles were replaced with flexible catheters and fixed with buttons. If applicator placement was judged insufficient during treatment planning, additional insertions were made.

### Brachytherapy

After applicator implantation, CT data were acquired with the patient in a supine position. CT slice thickness was < 3.0 mm. CT-based treatment planning was performed with the aid of Oncentra or PLATO (Nucletron BV, Veenendaal, The Netherlands). The high-dose-rate (HDR)-ISBT protocol was performed with microSelectron-HDR V2 using ^192^Ir as the source for monotherapy.

**Fig. 1:**
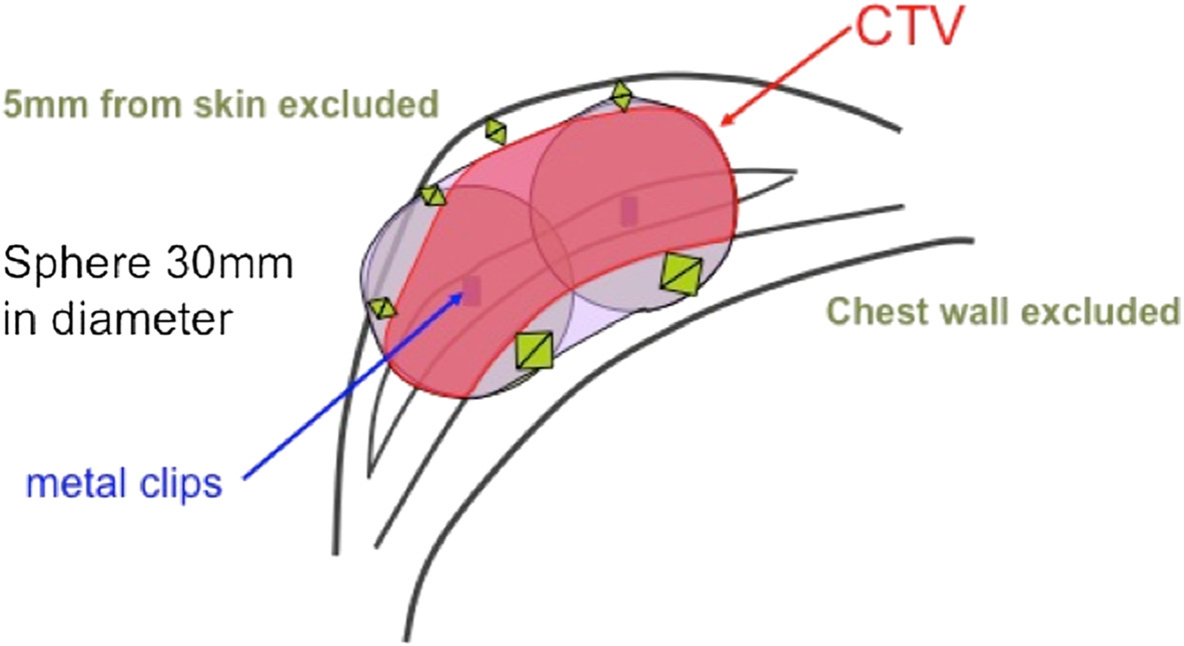
Clinical target volume (CTV) defines method

The clinical target volume was defined by drawing 3-cm diameter circles around each clip, and was considered to be the domain to which they were clinically connected (Fig. 1). The breast muscle layer and tissue of up to 5 mm depth from the skin surface were excluded. In addition, non-mammary gland tissue domain falling under CTV, identified by a consensus of the radiation oncologist and the breast surgeon, was excluded from CTV. The initial dose distribution was made using the Paris dosimetry system, which was modified by manual geometrical optimization when the skin dose was reduced. The brachytherapy was initiated the next day after applicator placement. A total dose of 36 Gy (six fractions of 6 Gy) was delivered as two fractions per day with an interval of 6 h between the two fractions.

### Dose specification

Compliance of each institution to the APBI protocol was assessed using three criteria:

1. Clip dose: The surgical clips in CTV were irradiated by >100 % of the isodose lines. A clip outside a mammary gland was excluded as an object. No exception was permitted.
2. Reference volume (Vref): The irradiated volume receiving >100 % of the prescription dose was 40-150 cm^3^. An exception was made for specific conditions (e.g., larger breast) under the approval of the study office and data center.
3. Dose non-uniformity ratio (DNR): The ratio was defined as the irradiated volume that received ≥ 1.5 times the reference dose over the volume that received ≥ 1.0 times the reference dose (DNR = V1.5ref/Vref). For all patients, DNR must be < 0.35, with no exception.

Compliance with the treatment plan was acknowledged when all three criteria were within the reference range.

### Quality assurance

To improve the quality of this study, workshops, rapid review, and Interim analysis were established. Quality assurance was implemented according to Radiation Therapy Oncology Group (RTOG) procedures [12]. Moreover, the data center predicted the total irradiation time from the Vref and source activity.

### Workshops

Three workshops were conducted to homogenize treatments between institutions. A radiation oncologist, a breast surgeon, a medical physicist, and a radiation technologist from each institution participated in all three workshops. The participants discussed the process of applicator insertion and the treatment plan. Moreover, each institution was instructed to submit their calculation of the benchmark treatment plan using uniform sample patient data, and the submitted data were verified at the data center.

### Rapid review

Each institution was required to fax a case report form (CRF) to the data center at least 24 h before the onset of treatment. The treatment plan summary contained no patient identifier. If there was any doubt, the data center queried the relevant institution and consulted with the study office. The data center and study office verified the following parameters: applicator length, offset value, clip dose, Vref, DNR, maximum skin dose, total treatment time, and source strength.

### Interim and final analyses

The number of patients required for the statistical analysis was determined on the basis of the optimal design of Simon [13]. Interim analysis was performed with data from 19 patients, and this clinical trial was planned to be stopped when non-compliance with the plan was confirmed for >3 patients. Final analysis was performed with data from 46 patients. The ISBT–APBI plan was defined as reproducible if non-compliance with the plan was confirmed for < 5 patients. The electronic data on the treatment plan from each institution was imported to Oncentra at the data center anonymously. The data center and study office reviewed all treatment plans.

## Results

### Patient demographics and treatment plan index

**Fig. 2:**
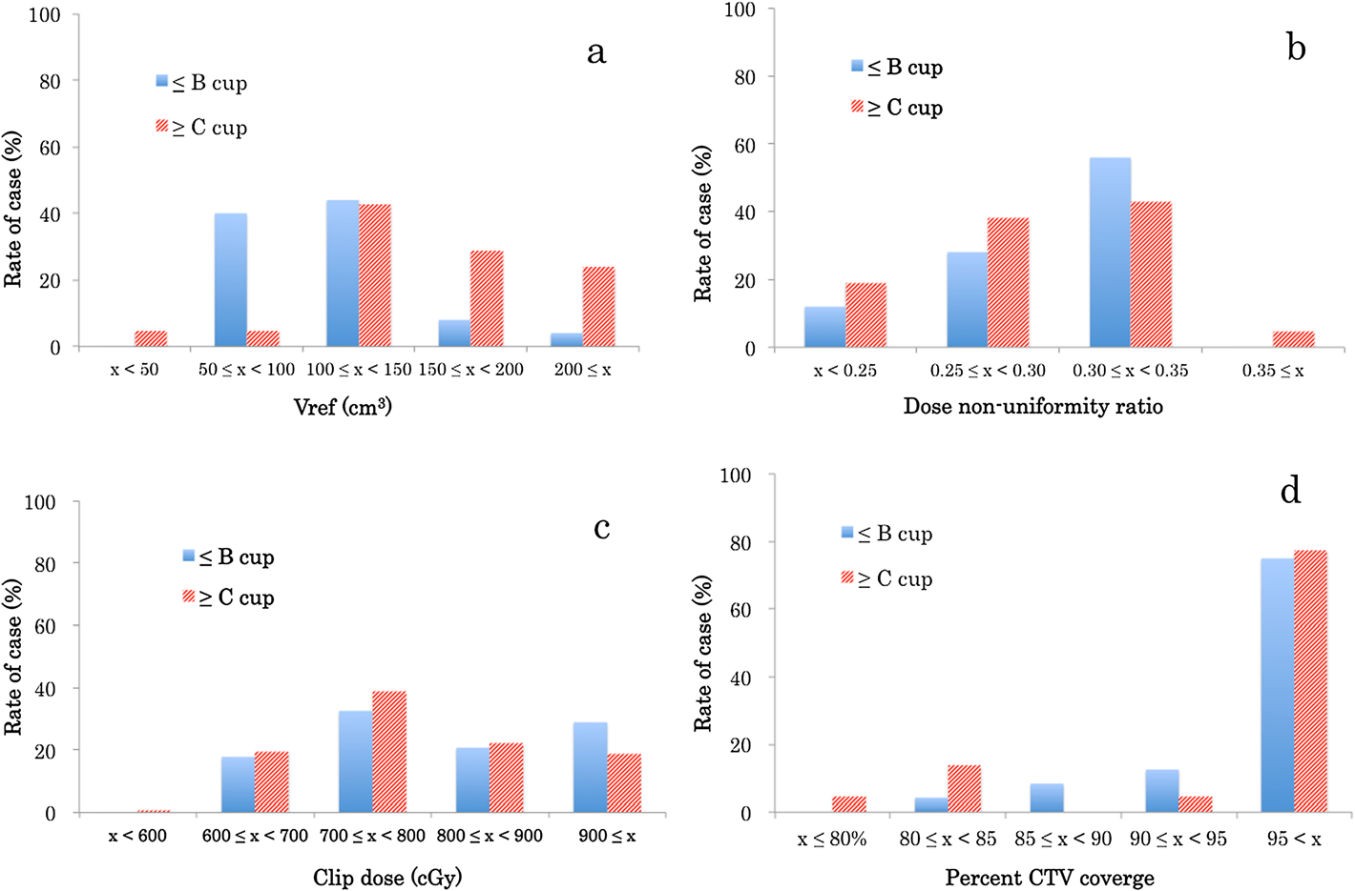
Frequency distribution of the index parameters of the ISBT–APBI treatment plan. **a** Reference volume (Vref). **b** Dose non-uniformity ratio (DNR). **c** Clip dose. **d** Percent CTV coverage

**Fig. 3:**
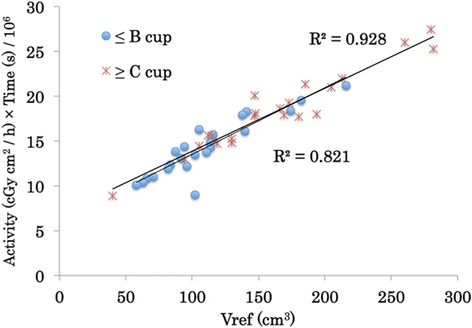
The relationship among activity, time and Vref. Activity is the activity the high-dose-rate source, time (s) is the total irradiation time, and Vref is the reference volume

The patient demographics are shown in Table 1. The patients were subjected to postoperative implantation by closed cavity (after breast-conserving surgery, *n* = 45) or perioperative implantation (during intra-breast-conserving surgery, *n* = 1). There were 24 patients (52 %) with a small bra size (A or B cup). Table 2 shows the index parameters of the treatment plan. The mean values of the dose-constrained parameters were as follows: Vref, 117 cm^3^ (range, 40-282), DNR, 0.30 (range, 0.22-0.51), and clip dose, 784 cGy (range, 469-3146). Fig. 2 shows the normal frequency distributions for Vref, DNR, clip dose, and percent CTV coverage among patients. The relationship among source activity, total irradiation time, and Vref are shown in Fig. 3. The correlation factors for in small bra size and large bra size patients were 0.821 and 0.928, respectively.

**Table 1:** Patient demographics

Variable	Mean ± SD (Range)	N (%)
Weight (kg)	60 ± 11 (43 – 96)	
Height (cm)	155 ± 6 (140 – 170)	
Body Mass Index	25.0 ± 4.8 (18.4 – 41.6)	
Number of catheters (n)	15 ± 3 (8 – 21)	
Breast excision weight (g)	82 ± 44 (25 – 234)	
Volume of CTV (cm^3^)	67 ± 29 (26 – 133)	
Bra size		
A cup		10 (22)
B cup		14 (30)
C cup		11 (24)
≥ D cup		11 (24)

**Table 2:** Index parameters of the treatment plan

Variable	Mean ± SD (Range)
Vref (cm^3^)	117 ± 56 (40 – 282)
DNR	0.30 ± 0.05 (0.22 – 0.51)
Clip dose (cGy)	784 ± 224 (469 – 3146)
Max skin dose (cGy)	521 ± 73 (223 – 607)
CTV V1.5ref (cm^3^)	39 ± 18 (12 – 98)
CTV V2.0ref (cm^3^)	15 ± 9 (5 – 58)
CTV V100 (cm^3^)	63 ± 27 (25 –129)
CTV V90 (cm^3^)	63 ± 28 (26 – 131)
CTV D100 (cGy)	565 ± 125 (146 – 624)
CTV D90 (cGy)	680 ± 97 (225 – 731)
CTV Mean dose (cGy)	927 ± 80 (760 – 1092)
Conformity Index	2.07 ± 0.84 (1.07 – 4.45)

### Validation of the reference volume

The data center and study office evaluated the adequacy of the Vref values that were outside the 40-150 cm^3^ range. In total, 14 patients had a Vref above the upper limit, with a median of 191 cm^3^ (range, 169-287). The patients with a Vref of > 150 cm^3^ had a median body mass index (BMI) of 28.3 (range, 25.5-41.6) and a median excision weight of 110 g (range, 60-234). In contrast, those with a Vref of ≤ 150 cm^3^ had a median BMI of 22.5 (range, 18.4-30.8) and a median excision weight of 61 g (range, 25-100). All exceeding Vref values were determined to be appropriate.

### Interim and final analyses

The interim analysis identified two non-compliant treatment plans. The analysis report was submitted to the safety monitoring committee, and clinical trial continuation was approved. In the final analysis, one more treatment plan was judged non-compliant with the protocol.

In case #1, the DNR value was 0.51, which was not within the acceptable limit (<0.35). This discrepancy was due to the large size of the patient’s breast (I cup). The target was larger than expected because the cavity was filled with fluid, which required several applicators. However, the patient refused the insertion of additional applicators. In case #2, the CTV creative method was not in accordance with the protocol. Not all 3-cm spheres created around the clips were connected. However, the dose distribution was connected, which was judged to be clinically satisfactory. In case #3, the one clip dose was 469 cGy, less than the reference value and wrong clip dose value was written in CRF. Thus, a total of 43/46 treatment plans were judged to be compliant and ISBT–APBI was concluded to be reproducible.

## Discussion

This study was designed to evaluate the reproducibility of ISBT–APBI for breast cancer patients among Japanese institutions. To our knowledge, this is the first reported ISBT–APBI multi-institutional clinical trial in Asia. In this study, ISBT–APBI was compliant for 43/46 patients. Although ISBT involves multiple manual procedures, standardization of the treatment plan is possible through comprehensive webinars. Two cases (case #2 and #3) deviated from the protocol due to a human error detected in CRF. Thus, careful review of the electronic data on the treatment plan is essential to secure the quality of a multicenter clinical trial.

**Fig. 4:**
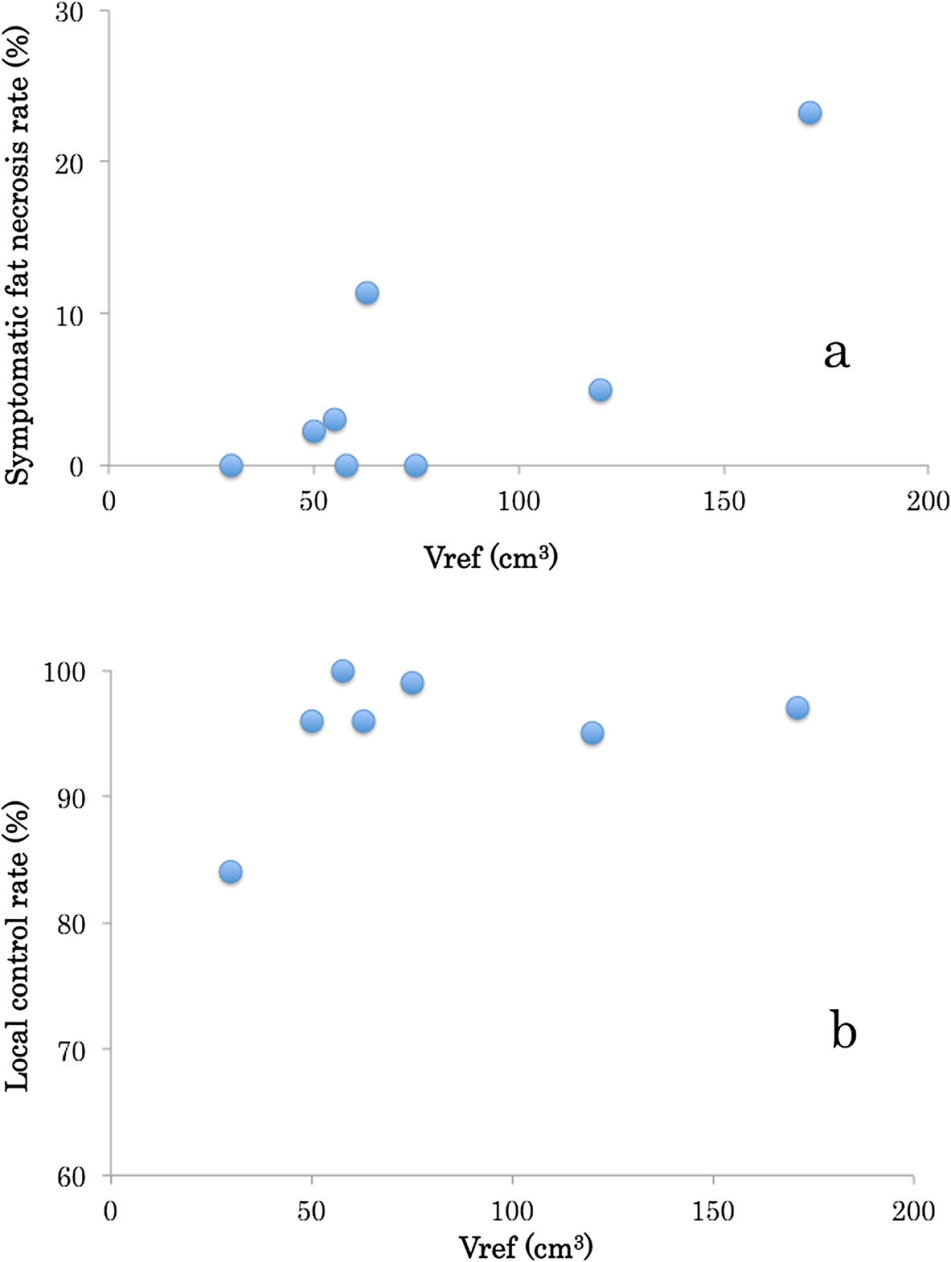
The relationship between irradiated volume, fat necrosis, and local control rate. **a** fat necrosis and Vref. **b** relationship local control rate and Vref

Based on preceding literature, two indices (Vref and DNR) of a brachytherapy plan are found to be important for local control and sequel. Figure 4 shows the relationship between irradiated volume, fat necrosis, and local control rate which have been reported by some authors [11, 14–21]. Wazer et al. reported that Vref, V1.5xref, and V2.0ref correlated with fat necrosis [16, 22]. Ott et al. concluded that irradiation volume correlated significantly with side-effects and local control, and a suitable upper limit of Vref was 150-180 cm^3^ for a closed cavity [9]. In contrast, Perera et al. reported a local control ratio of 84 % in an irradiated volume of 30 cm^3^ [17]. In literature from Germany and Austria, the Vref range was 48-84 cm^3^ [9, 18–20]. The protocol of the Phase III multi-institutional clinical trial of the Groupe Européen de Curiethérapie*-European* Society for Therapeutic Radiology and Oncology (GEC-ESTRO) adopted a Vref range of 40-150 cm^3^ with a DNR of < 0.35 [10]. In USA, the rate of open cavity breast surgery and the radiation volume are larger than those in Europe and Japan. Thus, the reference indicators of The National Surgical Adjuvant Breast and Bowel Project (NSABP)/RTOG 0413 [8, 10] may not apply to other continents.

Most patients with excessive Vref had a pyknic body type and required large-weight excisions. Moreover, six (13 %) patients had a BMI of > 30, corresponding to a 3.2 % deviation (Organization for Economic Co-operation and Development Health data 2009 [23]) from the general Japanese female population. There is a possibility that the APBI plan deliberately selected large-breast patients, because most Japanese females have small breasts and insertion of the applicator is difficult. This ISBT–APBI plan provided a better coverage of the target area, because a median 98 % of CTV received 100 % of the prescription dose, compared to 96 % of the single-institutional study by Das et al. [24].

The quality assurance methods of HDR treatment planning have been reported [24–26]. One of a quick, easy way of checking is a method to infer the total irradiation time from the source activity and the Vref. According to our results, there is low correlation among the Vref, the source activity and the total treatment time in the small bra-size treatment plan compared with that in the large bra-size treatment plan (Fig. 3). For small bra size patients, applicator insertion is difficult and the applicator spacing tends to be irregular and narrow. Moreover, each point source stop time in the small bra-size patient treatment plan is short compared with that in the large bra-size patient. The Vref increases exponentially with increasing source stop time because the dose distribution expands concentrically. Therefore, the method of inferring total irradiation time from the source activity and Vref is difficult to adjust to patients with small breasts.

## Conclusions

This prospective study defined an ISBT–APBI treatment plan to be clinically feasible for most Asian females with a closed cavity and small breast size. Furthermore, the treatment plan was reproducible between institutions, despite various levels of skills and experience with the technique.
